# Effects of Sohamhyoong-Tang on Ovalbumin-Induced Allergic Reaction in BALB/c Mice

**DOI:** 10.1155/2016/6286020

**Published:** 2016-06-14

**Authors:** So Hyun Jo, Yun Jung Lee, Dae Gill Kang, Ho Sub Lee, Dae Ki Kim, Min Cheol Park

**Affiliations:** ^1^Department of Oriental Medical Ophthalmology & Otolaryngology & Dermatology, College of Oriental Medicine, Wonkwang University, 460 Iksandae-ro, Iksan, Jeonbuk 54538, Republic of Korea; ^2^College of Oriental Medicine and Professional Graduate School of Oriental Medicine, Wonkwang University, 460 Iksandae-ro, Iksan, Jeonbuk 54538, Republic of Korea; ^3^Hanbang Body-Fluid Research Center, Wonkwang University, 460 Iksandae-ro, Iksan, Jeonbuk 54538, Republic of Korea; ^4^Department of Immunology and Institute of Medical Sciences, Chonbuk National University Medical School, Jeonju, Jeonbuk 54896, Republic of Korea

## Abstract

IgE-mediated mast cell degranulation and excessive Th2 cells activation are major features of various allergic diseases.* Sohamhyoong-tang* has been reported to have anti-inflammatory and antibacterial effects. In this study, we investigated the inhibitory effect of* Sohamhyoong-tang* extract (SHHTE) on allergic symptoms and inflammatory responses in ovalbumin- (OVA-) sensitized BALB/c mice. The mice were sensitized with OVA and alum at 2-week intervals and then orally given SHHTE for 13 days followed by intradermal OVA injection. Administration of SHHTE significantly reduced edema formation and inflammatory-cell infiltration in ear tissues. Total and OVA-specific IgEs as well as proinflammatory cytokine TNF-*α* and Th2-associated cytokine IL-4 levels were lower in the SHHTE-treated group than in the vehicle. SHHTE treatment significantly suppressed both mRNA and protein levels of IL-4 and IL-5 in OVA-stimulated splenocytes. SHHTE decreased Th1 (IFN-*γ*) and Th17 (IL-17a) cytokine mRNA expression but increased Treg cytokines (IL-10 and TGF-*β*1). Moreover, SHHTE significantly inhibited degranulation of RBL-2H3 cell line in a dose-dependent manner. Thus, SHHTE efficiently inhibited the allergic symptoms in an OVA-sensitized mouse model and its action may correlate with the suppression of IgE production by increasing IL-10 and TGF-*β*1, which can limit the function of other T helper cells and prevent the release of inflammatory mediators from mast cells. These results suggest that SHHTE could be a therapeutic agent for treating various allergic diseases.

## 1. Introduction

Allergy is a concept that was initially introduced in 1906 by a pediatrician named Clemens, which meant harmful reactions of the host against foreign materials [1]. Recently, allergy refers to an acute-type hypersensitivity reaction as an immune response that occurs within minutes after IgE antibodies attached to the surface of mast cells interact with antigens, which is the most common form of sensitization that occurs in 20% of the entire population [2, 3]. In allergic diseases, histamine, TNF-*α*, and IL-1 released from mast cells by IgE-mediated inflammatory reactions and activation of Th2 cells, a type 2 helper T cell, which secrete a variety of proinflammatory cytokines such as IL-4, IL-5, and IL-13 play an important role and besides the roles of regulatory T cells and helper T cells such as Th1 and Th17 are involved in the regulation of allergic inflammatory reactions [4, 5].

Allergic inflammatory reactions can be classified in various ways according to the organs that they appear in, among which urticaria, angioedema, atopic dermatitis, and allergic contact dermatitis are classified as skin diseases [6, 7]. Complementary and alternative medicine (CAM) has been a popular treatment modality among allergic disease patients. In a study of the prevalence of CAM use, more than one-third of respondents reported using CAM [8].

*Sohamhyoong-tang* is a prescription listed first in* Shang Han Lun*, which was composed of* Coptidis Rhizoma*,* Pinelliae Rhizoma*, and* Trichosanthis Semen* and has been used for pneumonia, pleuritis, chronic bronchitis, and pain in the midriff in Korea and China. Existing experimental studies on* Sohamhyoong-tang* include an anti-inflammatory effect on mouse macrophages, antibacterial activities, an antihyperlipidemia effect, and an anti-inflammatory analgesic effect [9–11]. Based on the comprehensive results of previous studies on* Sohamhyoong-tang*, an inhibitory effect for allergic inflammatory reactions is likely to be expected, but the relevant study has not been conducted yet.

Thus, the aim of this study was to investigate the antiallergic effect of* Sohamhyoong-tang*, and here we report the significant results that we obtained, which demonstrate the effect of* Sohamhyoong-tang* on allergic inflammatory reactions by oral administration of* Sohamhyoong-tang* to an allergic mouse model using OVA as an antigen, followed by measuring allergic skin reactions, infiltration of inflammatory cells within tissues, total amount of IgE and IgG1 secretion in the serum, inflammatory cytokines in the serum, and degranulation of mast cells.

## 2. Materials and Methods

### 2.1. Preparation of SHHTE

The 3 herbal ingredients of SHHTE were purchased from the Wonkwang Iksan Oriental Medical Hospital (Iksan, Korea). The ingredients of SHHTE include* Coptidis Rhizoma* (4 g),* Pinelliae Rhizoma* (12 g), and* Trichosanthis Semen* (4 g). Total SHHTE (200 g) was boiled with 1.8 L of distilled water at 100°C for 2 h. The resulting extract was filtered through Whatman No. 3 filter paper and centrifuged at 990 ×g for 20 min at 4°C. The resulting supernatant was concentrated using a rotary evaporator, after which the resulting extract (20.56 g) was lyophilized using a freeze-drier and stored at −70°C until it is required.

### 2.2. Experimental Animals

All experimental procedures were carried out in accordance with the National Institutes of Health* Guide for the Care and Use of Laboratory Animals* and were approved by the Institutional Animal Care and Utilization Committee for Medical Science of Chonbuk National University (CBU2014-00052). OVA-sensitized mice model was described by Park et al. [12]. Female 8-week-old BALB/c mice were obtained from Central Lab. Animal Inc. (Seoul, Korea) and immunized intraparenterally with 20 *μ*g of ovalbumin (OVA, grade V, Sigma, St. Louis, MO) and 2 mg aluminum hydroxide (alum, Thermo Scientific, Cramlington, UK) gel suspended in 100 *μ*L saline. On the 14th day, the mice were given an intraperitoneal booster injection of the same antigen or alum. All mice were randomly divided into five groups (*n* = 7) as follows: control, OVA-sensitization, OVA + 50 mg/kg/day SHHTE, OVA + 100 mg/kg/day SHHTE, and OVA + 0.5 mg/kg/day dexamethasone. SHHTE or dexamethasone was administered orally by sonde for a period of 13 days after 2nd sensitization and then blood was collected from the retroorbital plexus. For the measurement of ear thickness, intradermal 20 *μ*L OVA (1 mg/mL) was injected in ear tissues on the final day. Ear thickness measurements were performed by thickness gage (Mitutoyo, Japan).

### 2.3. Histological Study

Mice were sacrificed 24 hr after sensitization in ear tissues. The auricular skin was removed, fixed in 4% paraformaldehyde, embedded into paraffin, cut in 4 *μ*m sections, and stained with hematoxylin-eosin solution. Each section was analyzed using light microscopy (CX21, Olympus, Japan).

### 2.4. Measurement of Immunoglobulin and Cytokine Levels

Serum samples were obtained by 3,000 rpm centrifugation for 10 min. Total IgE and IgG1 levels were measured by mouse IgE ELISA kit and mouse IgG1 ELISA kit, respectively (BD Biosciences, San Jose, USA). OVA-specific IgE and IgG1 levels were measured by mouse anti-OVA IgE and anti-OVA IgG1 ELISA kit (Shibayagi, Japan). Cytokine levels were measured by sandwich mouse TNF-*α*, IL-4 ELISA kit (BD Biosciences, San Jose, USA). The levels of IL-4 and IL-5 for the Th2 cytokines in splenocytes medium were measured using IL-4 and IL-5 ELISA quantitation kits (BD Biosciences). The OD (optical density) was determined by using a microplate reader (Zenyth 200rt, Anthos, Austria).

### 2.5. Preparation of Splenocytes

Splenocytes were harvested as described by Trop et al. [13]. The spleen was removed at the time of killing and a single-cell suspension was prepared by forcing spleen through a 100 *μ*m stainless steel mesh. Erythrocytes were lysed with hypotonic buffered solution and lymphocytes were washed with PBS prior to being resuspended in RPMI 1640 medium. Histopaque 1119 (Sigma Diagnostics, St. Louis, MO) was slowly placed underneath the cells suspended and centrifuged at 2,000 rpm for 30 min at room temperature. Cells at the interface were collected, diluted in a 50 mL tube, and washed twice with ice-cold PBS (1,200 rpm for 10 min). The viability by trypan blue staining was more than 95%. Viable cells were counted by the trypan blue exclusion method using a hemocytometer. Finally, cells were adjusted into different concentrations with 10% FBS and RPMI 1640 medium.

### 2.6. RNA Isolation and Real-Time qRT-PCR

A kit from Qiagen (RNeasy*™* Plus Mini Kit) was used for RNA isolation from cell cultures, and RNA quality was tested by measuring the ratio 260/280 nm in a UV-spectrophotometer. Real-time qRT-PCR analysis was optimized by SYBR Green 2-step qRT-PCR kit protocol (DyNAmo*™*, Finnzymes, Finland). Specific primers were as follows, respectively: IL-4, sense: ATGGGTCTCAACCCCCAGCTA-3, antisense: TGCATGGCGTCCCTTCTCCT;  IL-5, sense: TGAAGGCCAGCGCTGAAGAC, antisense: GCGGACAGCTGTGTCAAGGTC; IL-13, sense: ATGAGTCTGCAGTATCCCG, antisense: CCGTGGCAGACAGGAGTGTT; IFN-*γ*, sense: AGCCAAGCGGCTGACTGAACT, antisense: TAAAGCGCTGGCCCGGAGT; IL-17A, sense: TCACCCTGGACTCTCCACCG, antisense: GTCCAGCTTTCCCTCCGCAT; IL-10, sense: GGCCCTTTGCTATGGTGTCCT, antisense: GTAGGGGAACCCTCTGAGCTGC; TGF-*β*1, sense: CCTGAGTGGCTGTCTTTTGA, antisense: CGTGGAGTTTGTTATCTTTGCTG; GAPDH, sense: CATGGCCTTCCGTGTTC, antisense: CCTGGTCCTCAGTGTAGC. The PCR was started at 95°C for 10 minutes (hot start) to activate the AmpliTaq polymerase, followed by 40 cycles of amplification (denaturation at 95°C for 15 seconds, annealing at 60°C for 1 min, extension at 72°C for 60 seconds, and plate reading at 60°C for 10 seconds). The temperature of PCR products was elevated from 65°C to 95°C at a rate of 0.2°C/1 sec, and the resulting data were analyzed by using the software provided by the manufacturer (Applied Biosystems Inc., Foster City, USA).

### 2.7. RBL-2H3 Culture and CCK-8 Assay

Basophilic leukemia cell line RBL-2H3 was purchased from ATCC and cultured in MEM medium containing 10% fetal calf serum and 100 U/mL penicillin and 100 *μ*g/mL streptomycin. RBL-2H3 was seeded at 1 × 10^4^/well in 96-well plate (100 *μ*L/well). CCK-8 assay was performed by CCK-8 kit (Dojindo, Kumamoto, Japan).

### 2.8. *β*-Hexosaminidase Assay

For detecting *β*-hexosaminidase activity, 100 *μ*L of the supernatant of each group was added to 96-well plate, plus 100 *μ*L of substrate, 5 wells for each test solution, plus 5 wells for the background solution [14]. The mixture was incubated at 37°C for 90 min, and then 150 *μ*L of stop solution was added to stop the reaction. The absorbance at 405 nm (OD) of each well was measured. The percentage of enzyme release was calculated using the following formula:

(1) Degranulation %=SS+P−ScontrolScontrol+Pcontrol×100,

where *S* = OD  of  cell  medium × dilution  ratio; *P* = OD  of  cell  lysate × dilution  ratio; *S*_control_ = OD  of  control  cell  medium × dilution  ratio; *P*_control_ = OD  of  control  cell  lysate × dilution  ratio.

### 2.9. Statistical Analysis

Results are expressed as SEM. Data were analyzed using GraphPad Prism software (version 5.0). Student's *t*-test was used to determine significant differences between groups. Results of *p* < 0.05 were considered statistically significant.

## 3. Results

### 3.1. The Effect of SHHTE on Allergic Edema

**Figure 1:**
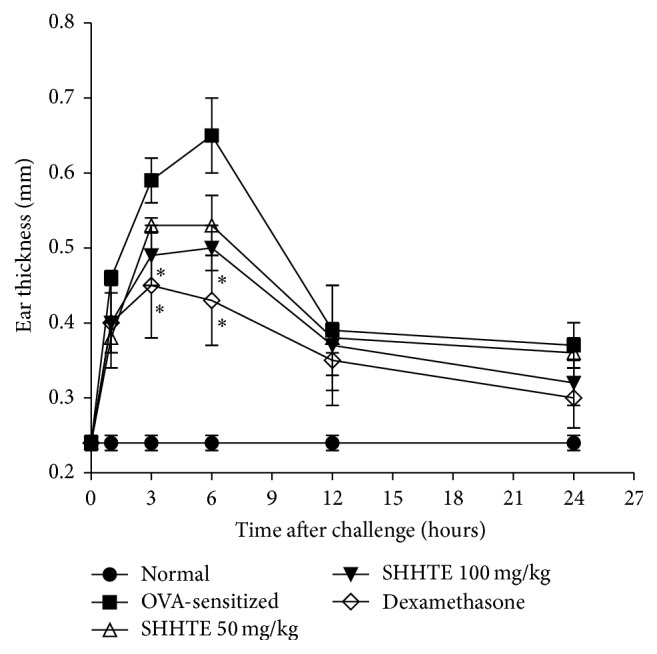
The effect of SHHTE on allergic cutaneous responses in OVA-sensitized mice. Data are given as means ± SEM (*n* = 7); ^*∗*^ *p* < 0.05 compared with OVA-sensitized group.

While ear thickness of the normal group was 0.24 mm on average, which showed no change over time, that of OVA-sensitization showed a tendency of an increase for 6 hours after the injection followed by the decrease afterwards. SHHTE group showed a reduced swelling reaction compared with OVA-sensitized group. In particular, OVA-sensitized group showed 0.59 mm (2.4-fold) and 0.65 mm (2.7-fold) increase after 3 and 6 hr, respectively. However, 100 mg/kg SHHTE administration showed 0.53 mm and 0.50 mm, respectively, which were 18% and 23% decreases, indicating that SHHTE significantly inhibited allergic skin reactions. Dexamethasone, the positive control, showed 0.45 mm and 0.43 mm, which were 24% and 34% decreases, respectively (Figure 1).

### 3.2. The Effect of SHHTE on the Morphological Changes of the Tissue Organization

**Figure 2:**
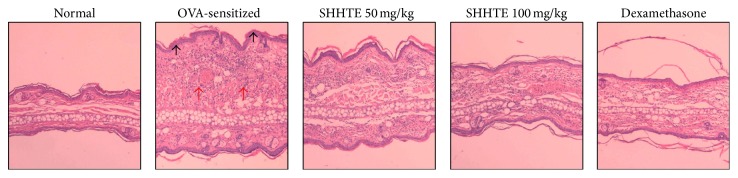
The effect of SHHTE on histological feature of ear tissues in OVA-sensitized mice. The red arrows indicate areas of inflammatory infiltrate and the black arrow indicates wheal response with underlying intradermal sensitization. Original magnification: 100x.

OVA-sensitization showed some characteristics such as a wheal response and increased infiltration of inflammatory cells after exposure to allergens compared with normal. On the other hand, SHHTE showed improved pathologic structures due to the reduction of the wheal response and infiltration of inflammatory cells at the inflammation area in a concentration-dependent manner. In particular, the degrees of swelling in the ear tissue and infiltration of inflammatory cells in 100 mg/kg SHHTE were reduced to nearly the same level as dexamethasone. Therefore, it was confirmed that administration of SHHTE reduced inflammatory reactions caused by exposure to allergens (Figure 2).

### 3.3. The Effect of SHHTE on the Content of Immunoglobulins in the Serum

When compared with the normal group, total IgEs and IgG1s of OVA-sensitization were significantly increased by 90% and 79%, respectively. Total levels of IgEs of 50 and 100 mg/kg SHHTE were decreased by 28% and 37%, respectively. Production of OVA-specific IgEs was inhibited in a concentration-dependent manner and thus reduced by 27% and 49% in 50 and 100 mg/kg SHHTE, respectively, compared with OVA-sensitization. There was no difference in OVA-specific IgG1s compared with OVA-sensitized group like total IgG1s. Dexamethasone showed a reducing effect (Table 1).

### 3.4. The Effect of SHHTE on the Content of Serum TNF-*α* and IL-4

**Figure 3:**
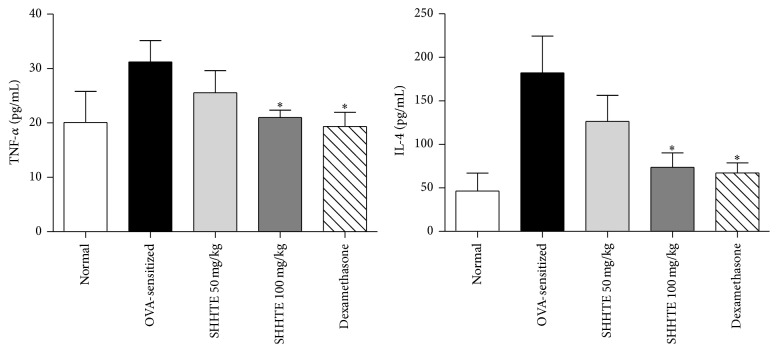
The effect of SHHTE on serum level of TNF-*α* and IL-4 in OVA-sensitized mice. Data are given as means ± SEM (*n* = 7); ^*∗*^ *p* < 0.05 compared with OVA-sensitized group.

The level of TNF-*α* was significantly increased to 31.21 ± 3.93 (pg/mL) in OVA-sensitization. 50 and 100 mg/kg SHHTE showed 27.31 ± 4.51 (pg/mL) and 20.99 ± 1.36 (pg/mL), respectively, indicating that TNF-*α* in the serum was reduced in a concentration-dependent manner. In particular, TNF-*α* in the serum was significantly decreased in 100 mg/kg SHHTE group to similar levels to Dexa group. SHHTE also decreased OVA-induced IL-4 production in a concentration-dependent manner (Figure 3).

### 3.5. The Effect of SHHTE on Th2 Cytokines IL-4, IL-5, and IL-13 in the Splenocytes of OVA-Sensitized Mice

**Figure 4:**
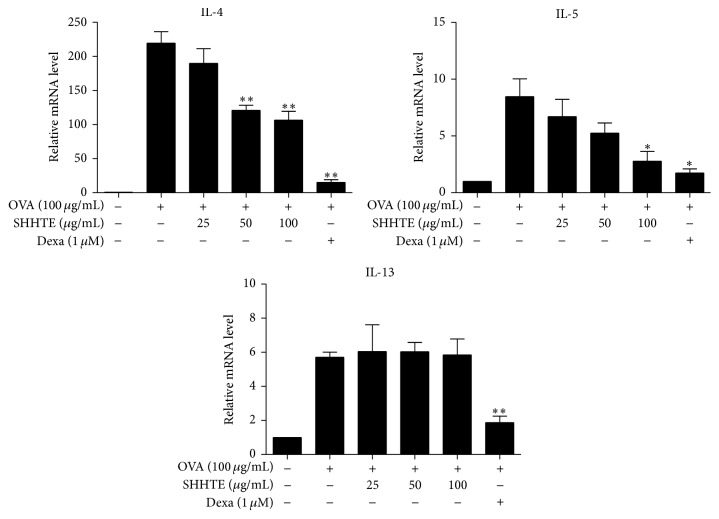
The effect of SHHTE on mRNA expression of Th2 cytokines in splenocytes isolated from OVA-sensitized mice. Cells were treated with various doses (25, 50, and 100 *μ*g/mL) of SHHTE with or without OVA in vitro. The mRNA expressions of IL-4, IL-5, and IL-13 were assessed by real-time PCR. Data are given as mean ± SEM of three independent experiments. ^*∗*^ *p* < 0.05 and ^*∗∗*^ *p* < 0.01 compared with control.

**Figure 5:**
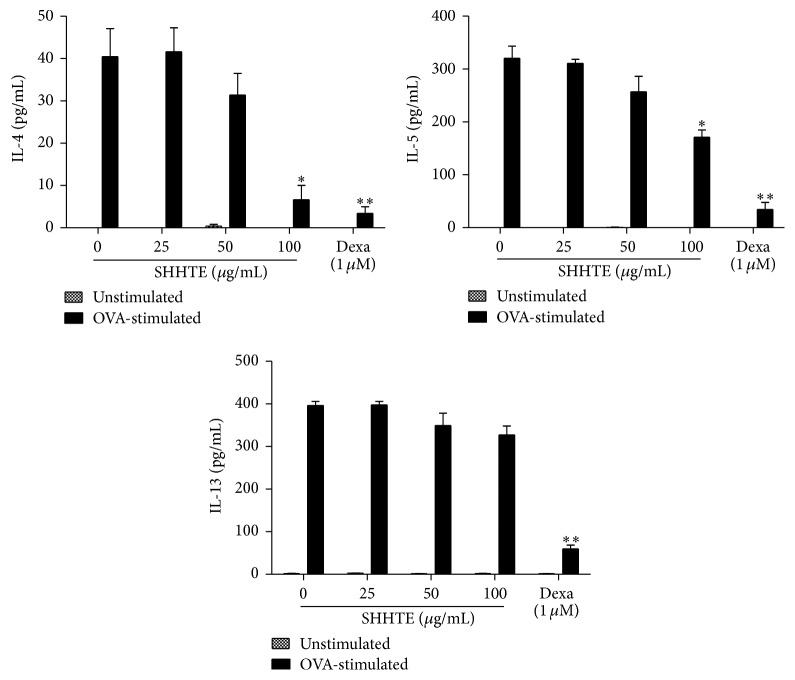
The effect of SHHTE on the secretion of Th2 cytokines in splenocytes isolated from OVA-sensitized mice. Cells were treated with various doses (25, 50, and 100 *μ*g/ml) of SHHTE with or without OVA in vitro. Secretions of Th2 cytokines IL-4, IL-5, and IL-13 were measured using ELISA kit. Data are given as mean ± SEM of three independent experiments. ^*∗*^ *p* < 0.05 and ^*∗∗*^ *p* < 0.01 compared with control.

Cytotoxicity test supposed that the concentration range of up to 100 *μ*g/mL SHHTE was used for cytokine experiments using splenocytes (data not shown). As shown in Figure 4, of the Th2 cytokines, mRNA expression of IL-4 showed about 219-fold increase with OVA and IL-5 and IL-13 also showed 8-fold and 6-fold increases with significance, respectively. mRNA expressions of IL-4 and IL-5 were decreased by administration of SHHTE in a concentration-dependent manner; however, IL-13 did not show any significant difference from OVA-treated group. The analysis of protein amount of each cytokine in the cell culture medium demonstrated that 100 *μ*g/mL SHHTE decreased the contents of IL-4 and IL-5 by 84% and 47%, respectively. Although IL-13 showed a trend of decrease, it did not show any significant difference from OVA-stimulated splenocytes (Figure 5).

### 3.6. The Effect of SHHTE on Th1 (IFN-*γ*) and Th17 (IL-17A) Cytokines in the Splenocytes of OVA-Sensitized Mice

**Figure 6:**
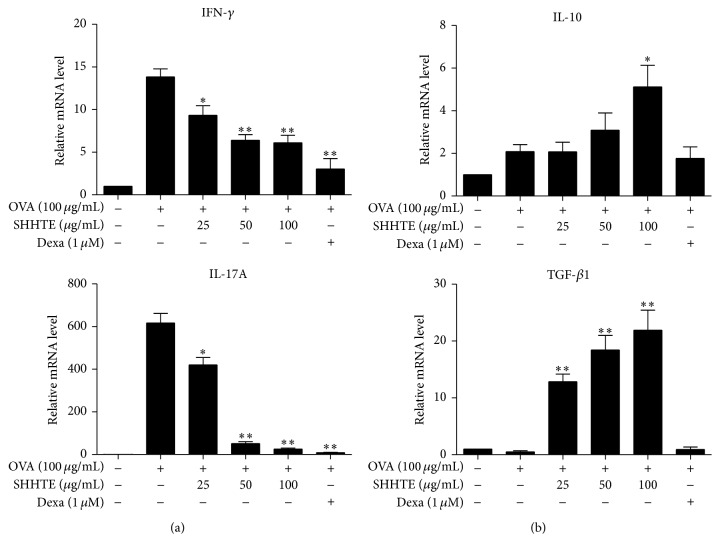
The effect of SHHTE on mRNA expression of Th1 (IFN-*γ*)/Th17(IL-17A) and Treg cytokines in splenocytes isolated from OVA-sensitized mice. Cells were treated with various doses (25, 50, and 100 *μ*g/mL) of SHHTE with or without OVA in vitro. The mRNA expression of IFN-*γ* and IL-17A (a) or IL-10 and TGF-*β*1 (b) was assessed by real-time PCR. Data are given as mean ± SEM of three independent experiments. ^*∗*^ *p* < 0.05 and ^*∗∗*^ *p* < 0.01 compared with control.

mRNA expression of IFN-*γ* which is a cytokine secreted by Th1 cells and IL-17A secreted by Th17 cells was significantly increased in the splenocytes of OVA-sensitized mice. mRNA expressions of IFN-*γ* and IL-17A were significantly reduced by SHHTE in a concentration-dependent manner (*p* < 0.01) (Figure 6(a)).

### 3.7. The Effect of SHHTE on Treg (IL-10, TGF-*β*1) Cytokines in the Splenocytes of OVA-Sensitized Mice

mRNA expressions of IL-10 and TGF-*β*1 which are immune-regulatory cytokines known to be secreted by Treg cells were increased in SHHTE treatment in a concentration-dependent manner. However, Dexa-treated group showed no significant increase in the mRNA expressions of IL-10 and TGF-*β*1 (Figure 6(b)).

### 3.8. The Effect of SHHTE on Degranulation of RBL-2H3 Cell Line

**Figure 7:**
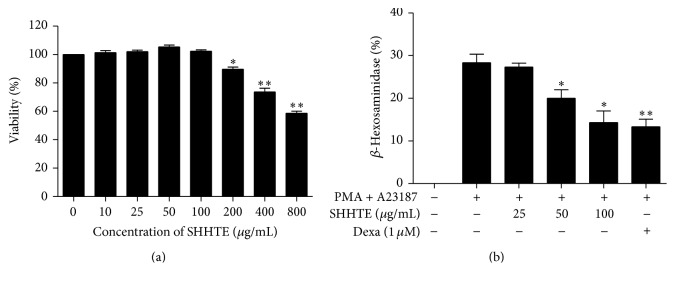
(a) Cytotoxicity of SHHTE in RBL-2H3 cell line. Cells were treated with various doses (10, 25, 50, 100, 200, 400, and 800 *μ*g/mL) of SHHTE and incubated for 24 hr. Cytotoxicity was determined using CCK-8 kit. Data are given as mean ± SEM of three independent experiments. ^*∗*^ *p* < 0.05 and ^*∗∗*^ *p* < 0.01 compared with control. (b) The effect of SHHTE on degranulation of RBL-2H3 cell line. Cells were treated with various doses (25, 50, and 100 *μ*g/mL) of SHHTE in the presence or absence of PMA (50 ng/mL) and A23187 (500 ng/mL). Data are given as mean ± SEM of three independent experiments. ^*∗*^ *p* < 0.05 and ^*∗∗*^ *p* < 0.01 compared with control.

After treatment with different concentrations of SHHTE, cells were incubated for 24 hours followed by measurement of absorbance, and the results showed no cytotoxicity up to 100 *μ*g/mL of SHHTE in RBL-2H3 cell line. Degranulation was induced by treatment of stimulants of phorbol 12-myristate 13-acetate (PMA), a PKC activator, and A23187, calcium ionophore. The discharge amount of *β*-hexosaminidase was significantly increased in the control group treated with the stimulants with about 28% being released, and degranulation in the cells is decreased in a concentration-dependent manner in SHHTE-treated group (Figure 7).

## 4. Discussion

In this study, we investigated the effect of traditional formula SHHTE on allergic responses by sensitizing BALB/c mice with OVA. IgEs are typically generated against allergens and mast cells and basophils in human and experimental animals have a strong affinity for the Fc portion of the IgE antibody. Once mast cells are activated when IgE antibodies attached to master cells and basophils bind to antigens, local vascular permeability is increased within a few minutes after exposure of these chemical mediators to the antigens, which leads to immediate allergic reactions causing edema and erythema. Late phase responses are typically triggered by accumulation of inflammatory cells such as eosinophils and neutrophils due to the inflammatory mediators and chemotactic factors secreted by master cells [15, 16].

The administration of SHHTE and OVA-exposure showed that allergic edema reactions were reduced over time and infiltration of inflammatory cells appears in the late phase. In order to investigate the effect of SHHTE on the content of immunoglobulins that are increased by allergic immune responses and trigger activation of cells by being attached to the surface of mast cells and basophils, total IgEs and IgG1s as well as OVA-specific IgEs and IgG1s were measured in the serum of the experimental animals. While total IgEs and OVA-specific IgEs were increased when the mice were sensitized to OVA antigen, they were reduced with administration of SHHTE in a concentration-dependent manner. SHHTE showed a more significant effect than dexamethasone, suggesting that SHHTE effectively suppressed IgE production caused by allergen sensitization. IL-4 secreted by Th2 cells is known to induce production of IgG1s as well as IgG isotype switching in B-lymphocytes in a mouse [17, 18]. The results of the analysis of total IgG1s and OVA-specific IgG1s in the serum of SHHTE-treated group showed no significant difference compared with OVA-sensitized group, suggesting that the suppressive effect of SHHTE was caused by its effect on production of IgE antibodies rather than IgG1s.

TNF-*α* is one of the cytokines secreted by mast cells, which is involved in allergic inflammatory reactions. It is known that, after antigen challenges to the atopic dermatitis tissues or sensitized individuals, expressions of adhesion molecules in the vascular endothelial cells such as E-selectin and ICAM-1 are increased and it was reported that the movement and migration of inflammatory cells are promoted by the increase of these cell adhesion molecules in the vascular endothelial cells [19, 20]. TNF-*α* in the serum was decreased in SHHTE-treated group in a concentration-dependent manner, which suggests that it influenced the suppression of the inflammatory responses which were induced after exposure to antigens.

If allergens enter the body, cytokines IL-4, IL-5, and IL-13 are secreted due to the induction of Th2 cell differentiation. In particular, IL-4 is known to play an important role in increasing production of IgEs from B cells [21]. We examined whether SHHTE reduced activation of Th2 cell differentiation and increased secretion of cytokines which are observed in allergic diseases. Splenocytes isolated from OVA-sensitized mice were cultured following OVA-stimulation. mRNA expressions of IL-4 and IL-5 were significantly reduced by SHHTE treatment in a concentration-dependent manner. Measuring IL-4 in the serum also showed a significant decrease in SHHTE-treated group, suggesting a possibility that IgE production and inflammatory responses were reduced by an inhibitory effect of SHHTE on Th2 cytokines. The effect of SHHTE on the allergic inflammatory reactions was not caused by reduced cell viability, but by unique properties of SHHTE.

Reactions of helper T cells caused by allergens are not limited only to the Th2 subtype and, thus, the importance of Th1 and Th17 cells is getting more attentions. IFN-*γ* secreted by Th1 cells was reported to be necessary for efficient induction of Th2 responses [22, 23] and IL-17A secreted by Th17 is also known to be an inflammatory cytokine which induces eosinophilic and neutrophilic inflammations in asthma and allergic rhinitis and plays a role in activating mast cells [16, 24]. mRNA expressions of a Th1 cytokine, IFN-*γ*, and a Th17 cytokine, IL-17A, were significantly increased by OVA-stimulation in the spleen cells, which was significantly decreased by SHHTE treatment. These results demonstrate that SHHTE suppressed allergic immune responses effectively by inhibiting secretion of cytokines from Th1/Th17 cells which are effector T cells induced by OVA as an antigen.

IL-10 and TGF-*β* secreted by Treg cells have a suppressive function for subtypes of other helper T cells and, currently, generating and maintaining allergen-specific Treg cells in the immunotherapy during allergy treatment are known to be important [25]. mRNA expressions of IL-10 and TGF-*β*1 were increased by cotreatment of OVA and SHHTE in a concentration-dependent manner. In contrast, dexamethasone did not show any significant difference. These results indicate that SHHTE suppressed production of Th2 cytokines by inducing expressions of IL-10 and TGF-*β*.

Considering that histamine and inflammatory mediators that are secreted during the activation of mast cells are the causes of allergic symptoms, discovery or development of mast cell stabilizers is one of the ways to treat allergies [26]. *β*-Hexosaminidase is contained in the granules within mast cells and basophils; thus, the degree of degranulation can be analyzed by measuring this enzyme in the cell culture media [27, 28]. To examine the effect of SHHTE on degranulation of mast cells and basophils, *β*-hexosaminidase released from RBL-2H3 cells, which is a rat-derived mast cell line, was measured. The activity of *β*-hexosaminidase was decreased by SHHTE in a concentration-dependent manner, leading to inhibition of degranulation.

The Sohamhyoong-tang consisted of three herbs, that is, Coptidis Rhizoma, Pinelliae Rhizoma, and Trichosanthis Semen. The main compound of Coptidis Rhizoma is berberine and it has well-known anti-inflammatory property in various types of experimental models [29–31]. There was no report for the anti-inflammatory effect of Pinelliae Rhizoma and Trichosanthis Semen. Thus, we cannot conclude whether a certain compound affects the treatment of allergic disease because no direct evidence exists on whether berberine improved allergy in an animal model such as OVA-sensitized Balb/c mice. Future study will clarify the standardization of Sohamhyoong-tang.

Taken together, SHHTE suppressed IgE production from B-lymphocytes not only by promoting expression of Treg cytokines such as IL-10 and TGF-*β*, which in turn suppressed Th2 cytokines (IL-4, IL-5) playing a critical role in the pathological mechanisms of allergies, but also by suppressing expressions of cytokines IFN-*γ* and IL-17A of Th1 and Th17 helper T cells, which worsens allergic reactions and prevents activation of T lymphocytes induced by allergens. In addition, it acted on immune cells in a variety of ways by inducing suppression of degranulation of mast cells or basophils, which led to improvement of allergic symptoms and inflammatory reactions. Therefore, we determined the inhibitory effect of SHHTE on allergic immune responses and suggested a potential application of Sohamhyoong-tang for prevention and treatment of IgE-mediated diseases and various allergic diseases.

## Figures and Tables

**Table 1:** The effect of SHHTE on serum level of total IgE and IgG1.

Groups	Total IgE (*μ*g/mL)	Total IgG1 (*μ*g/mL)	OVA-specific IgE (*μ*g/mL)	OVA-specific IgG1
Normal	1.43 ± 0.47 (10)	678.09 ± 70.48 (21)	ND	ND
OVA-sensitized	13.69 ± 1.26 (100)	3214.00 ± 32.89 (100)	97.57 ± 15.82 (100)	1745.15 ± 316.66 (100)
SHHTE 50 mg/kg	9.82 ± 0.72 (72)^*∗*^	3242.57 ± 76.03 (101)	71.29 ± 19.08 (73)	1559.35 ± 167.97 (89)
SHHTE 100 mg/kg	8.50 ± 0.74 (62)^**^	3221.01 ± 53.33 (100)	49.94 ± 12.05 (51)^*∗*^	1546.48 ± 241.31 (89)
Dexamethasone	9.37 ± 0.66 (68)^*∗*^	2856.91 ± 140.44 (89)^*∗*^	39.80 ± 10.02 (41)^*∗∗*^	833.49 ± 89.26 (48)^*∗*^

ND: not detectable. Data are given as means ± SEM (*n* = 7). ^*∗*^ *p* < 0.05 and ^*∗∗*^ *p* < 0.01 compared with OVA-sensitized group.
